# Effectiveness of a respectful maternity care program in a Guatemalan indigenous region rural hospital: a quasi-experimental study

**DOI:** 10.3389/fgwh.2025.1640952

**Published:** 2025-11-12

**Authors:** Hina Ikezoe, Shigeko Horiuchi, Modesta Girón

**Affiliations:** 1 Graduate School of Nursing Science, St. Luke’s International University, Tokyo, Japan; 2 Hospital Nacional de Joyabaj, Quiché, Guatemala

**Keywords:** respectful maternity care, mistreatment, childbirth, quality of care, indigenous, Guatemala

## Abstract

**Background:**

Mistreatment of women during childbirth in healthcare facilities can serve as a barrier to utilizing healthcare services. Respectful maternity care (RMC) has been recommended to address this issue, and interventions to promote RMC have been implemented globally. For Indigenous women in particular, such care is closely related to childbirth satisfaction and is considered crucial. However, research on RMC in Guatemala is limited, with no reports focusing on educational interventions. Therefore, this study aimed to implement an educational program to promote RMC for nurses and evaluate its effectiveness.

**Methods:**

This study employed a quasi-experimental design and was conducted at a hospital in the Quiché Department, Guatemala. For nurses in the hospital, a two-day educational program on RMC, which included lectures and group work, was implemented. The effectiveness of the program was assessed by comparing women's experiences of RMC and mistreatment during childbirth before and after the intervention. Data was analyzed using chi-square tests, independent *t*-tests, and ANCOVA.

**Results:**

This study included 176 postpartum women, with 88 in each pre- and post-intervention group. The average RMC scores significantly increased from 33.74 pre-intervention to 56.70 post-intervention (*p* < .001), representing a 68% relative increase. In the pre-intervention group, 71.6% of women experienced physical abuse, verbal abuse, or stigma or discrimination, which significantly decreased to 33.0% in the post-intervention group (*p* < .001).

**Conclusion:**

This educational program suggested improvements in women's childbirth experiences in the facility. Implementing this program in other facilities and regions could contribute to the widespread promotion of RMC practices in healthcare settings.

## Introduction

Since the 2010 report by Bowser and Hill (1), research and intervention studies on mistreatment during childbirth have been conducted globally. A study conducted across four countries worldwide reported that approximately one-third of women experienced some form of mistreatment during childbirth (2). Such mistreatment is a violation of women's fundamental human rights (3) and has been reported to be associated with barriers to the utilization of healthcare services (4), as well as potential adverse effects on mental health during the postpartum period (5–7), highlighting the urgency of addressing this issue. The World Health Organization (WHO) has stressed the importance of preventing and eliminating mistreatment during facility-based childbirth and recommends Respectful Maternity Care (RMC) (8). RMC is defined as “*care organized for and provided to all women in a manner that maintains their dignity, privacy, and confidentiality, ensures freedom from harm and mistreatment, and enables informed choice and continuous support during labour and childbirth (p19),”* and is recommended to promote positive childbirth experiences (9). Interventions to promote RMC have been shown to increase women's experiences of RMC and reduce experiences of mistreatment (10–12). However, these interventions have primarily been conducted in Africa and Asia, with limited research and reports in Latin America.

Latin America is the only region where the maternal mortality ratio (MMR) did not experience a significant decline from 2000 to 2020 (13). Maternal mortality is influenced by complex economic, cultural, and environmental factors, with social inequalities such as racism, poverty, gender inequality, and lack of education exacerbating these impacts (14). Specifically, Guatemala is among the countries with the highest MMRs in the region (15) and has one of the largest Indigenous populations, comprising 43.6% of the total population (16, 17), with severe ethnic inequality. Indigenous people are predominantly rural residents facing high poverty rates, low educational levels, and limited access to healthcare (17, 18). In the context of sexual and reproductive health, Indigenous women have lower rates of facility-based childbirth and higher MMRs compared to non-Indigenous women (19–22). Barriers to healthcare access for Indigenous women include discrimination, mistreatment, language barriers, and a lack of culturally appropriate care (23–28). Studies conducted in Indigenous areas of Guatemala have reported various forms of mistreatment experienced by women during childbirth in healthcare facilities (23, 24). These findings emphasize the critical need to address mistreatment during childbirth in healthcare facilities in Guatemala.

In Guatemala, research on promoting RMC has been limited to the introduction of obstetric care navigators trained to provide comprehensive patient support (29), with no interventions specifically targeting improvements in hospital care. Given reports of inappropriate behaviors, such as verbal abuse and discrimination by healthcare providers (23–25), educational interventions targeting healthcare providers are essential. Educational interventions on RMC can enhance healthcare providers’ knowledge and awareness, reduce mistreatment experiences, and improve communication between women and healthcare providers (11). Thus, this study aimed to implement an educational program for nurses and evaluate its effectiveness. The following indicators were used to assess the intervention's effectiveness: the primary outcome was women's experiences of RMC during childbirth, and the secondary outcome was women's experiences of mistreatment during childbirth.

## Materials and methods

### Study design and setting

This quasi-experimental study with two non-equivalent groups used a pre-post-study design. An educational intervention was implemented for nurses at a hospital, and its effectiveness was assessed by comparing women's experiences of RMC and mistreatment during childbirth before and after the intervention. Since childbirth cannot be repeatedly experienced by the same woman within a short period, this study employed non-equivalent samples with matched participant characteristics. In addition, nurses who received the intervention were not informed of its contents in advance, and the participating women were not provided with any information about it.

This study was conducted at a hospital in the Quiché Department, Guatemala, where 89.2% of the population is Indigenous, one of the highest proportions in the country (17). The MMR in this region has consistently been high and was the highest in Guatemala in 2021 (22). The hospital is one of four operated by the Ministry of Public Health and Social Assistance in the Quiché Department. It provides various medical services, including internal medicine, surgery, obstetrics/gynecology, pediatrics, emergency, sexual violence clinic, psychology, and clinical laboratory.

### Study participants, recruitment, and sampling

Postpartum women of reproductive age (15–49 years) who had given birth at the hospital and spoke either Spanish or K'iche’ were eligible. Adolescents under 18 years of age were included if they had obtained consent through a proxy. Women who had experienced a stillbirth were excluded. To ensure similar group characteristics between the two groups, participants in the post-intervention group were recruited to match those in the pre-intervention group based on age, parity, and ethnicity. Potential participants were selected by the lead researcher, who had been engaged in the study setting for over five years in collaboration with nurses in the hospital. Due to the unpredictability of delivery timing and the limited data collection period, eligible women were recruited consecutively. The screening process involved identifying women who met the inclusion criteria based on delivery records. Women who met the criteria were invited to participate by the lead researcher or research assistants (RAs), who were Indigenous nurses fluent in both Spanish and K'iche’ and not employed at the hospital.

The sample size was determined using G*Power software version 3.1, based on an effect size of 0.46 derived from the study by Afulani et al. (30), with a power of 0.80 and an alpha level of 0.05. Accounting for a 20% anticipated dropout rate, the final sample size was calculated to be 176 women, with 88 women in each group.

### Description of intervention

The educational program promoting RMC was implemented to enhance nurses’ perception of RMC and raise awareness of mistreatment. The program was designed using the ADDIE instructional design model (31) and developed using existing literature and the women's narratives from the study by Ikezoe and Horiuchi (23). It consisted of two 3-hour sessions focused on mistreatment and RMC. The program combined face-to-face lectures and group work, emphasizing participatory learning through case studies and role-playing. The first session was conducted in April 2024, and the second in May 2024, with two sessions scheduled per month. Of the 50 participants, 12 were professional nurses, and 38 were auxiliary nurses. A 5-member team consisted of a head nurse, two professional nurses involved in nursing education, a social worker, and the lead researcher delivered the program following several preparatory meetings and rehearsals. After each session, participants were provided with lunch (50 GTQ per meal, approximately 6.42 USD).

### Data collection and management

Data for the pre-intervention group was collected from February to March 2024, while data for the post-intervention group was collected from May to July 2024. Data collection occurred when participants were discharged from the hospital. Participants who agreed to participate were escorted to a private room, where the lead researcher or the RAs explained the study again using relevant documents and obtained written informed consent. Data was collected via self-administered questionnaires or interviews. Participants with at least a high school education who chose to complete the Spanish-language questionnaire independently were given instructions on how to respond, and they filled it out independently after the researchers left the room. For all other participants, data was collected through interviews during which questions were read aloud in Spanish or played back in K'iche’ from a recording, after which they provided oral responses. RAs serving as interpreters received multiple training sessions, and the lead researcher attended the interviews to record responses. In addition, participants were informed that neither the lead researcher nor the RAs were affiliated with the hospital staff, and that their information would be kept confidential.

### Measurements and instruments

The primary outcome, women's experiences of RMC, was measured using the Person-Centered Maternity Care (PCMC) scale, which includes three subscales: dignity and respect, communication and autonomy, and supportive care (32). The PCMC scale is a 30-item, four-point Likert scale (0 = no, never; 1 = yes, a few times; 2 = yes, most of the time; and 3 = yes, all the time). Scores range from 0 to 90, with low scores indicating poor PCMC. The scale has been validated in Kenya, India, and Ghana, demonstrating high validity and good reliability (Cronbach's α = 0.85) (32–34).

The secondary outcome, women's experiences of mistreatment, was assessed using a brief version of the Community Survey Tool (CST) (35). The original CST, developed by Bohren et al. (36), contains over 70 items, which were considered difficult for postpartum women to answer. Therefore, a brief version with 22 items, demonstrating high agreement with the original tool, was used in this study. The brief version includes five subcategories: physical abuse, verbal abuse, failure to meet professional standards of care, poor rapport between women and providers, and health system conditions and constraints. The subcategory of stigma and discrimination included in the original tool was excluded from the brief version. However, four items related to stigma and discrimination, as reported in the previous study conducted at the hospital (23), were included, resulting in 26 items. Additionally, internal consistency was assessed after data collection, confirming adequate reliability (Cronbach's *α* = 0.80). Each item had three options (yes, no, or unknown) or five (strongly agree, agree, neutral, disagree, strongly disagree, and unknown). Both scales were translated from English to Spanish and then from Spanish to K'iche’, using forward and backward translation methods. Since K'iche’ is primarily a spoken language, the translated items were recorded as audio data. After the translation, local nurses reviewed both versions and made minor corrections. Demographic data was also collected, including age, parity, ethnicity, religion, education, occupation, marital status, economic status, language spoken at home, Spanish language proficiency, mode of birth, and birth experiences in facilities.

### Statistical analysis

The data was analyzed using SPSS version 30. Chi-square tests were used to compare the participants’ sociodemographic characteristics between the groups. For women's experiences of RMC, full and subscale scores on the PCMC scale were calculated. The normality of the distribution was verified, and independent *t*-tests were used to assess the differences in mean scores between the groups. An analysis of covariance (ANCOVA) was performed to adjust for potential confounders and compare score differences. For women's experiences of mistreatment, the percentages of women who reported experiencing each item were calculated. The percentage of individuals who experienced at least one item within the subcategories of physical abuse, verbal abuse, or stigma and discrimination was calculated, and these individuals were categorized as having experienced mistreatment. The comparison of groups on each item of the PCMC and mistreatment scales was performed using chi-square tests. A *p*-value of 0.05 or less was considered statistically significant.

### Ethical considerations

This study was approved by St. Luke's International University Research Ethics Committee (approval number 23-A099) and the study hospital in Guatemala. Participants were provided with explanations using written documents, and written informed consent was obtained from all participants. For participants under 18, written informed consent was also obtained from their parents or guardians. For illiterate participants, the study explanation was read aloud, oral consent was obtained, and they signed the consent form in writing or by fingerprint.

## Results

### Sociodemographic characteristics of participants

The detailed sociodemographic characteristics of the participants are presented in Table 1. The average age was 24.56 years (*SD* = 6.72) in the pre-intervention group and 23.38 years (*SD* = 6.43) in the post-intervention group, and around 40% were primiparous. Most participants were Indigenous, had less than a primary education, were married or cohabiting, and spoke the local language at home. The only statistically significant difference between the groups was in Spanish language proficiency (*p* = .031), with 29.5% of the pre-intervention group and 44.3% of the post-intervention group being unable to communicate in Spanish (either unable to speak or unable to listen or able to listen but not speak).

**Table 1:** Sociodemographic characteristics of the participants .

Characteristics	Pre-intervention (*n* = 88)		Post-intervention (*n* = 88)		*p*
	*n*	%	*n*	%	
Age (years)					
15–19	26	29.5	26	29.5	.134^a^
20–24	25	28.4	37	42.0	
25–29	13	14.8	11	12.5	
30–34	16	18.2	6	6.8	
≥35	8	9.1	8	9.1	
Parity					
1	36	40.9	36	40.9	.255^a^
2	26	29.5	21	23.9	
3	9	10.2	18	20.5	
≧4	17	19.3	13	14.8	
Ethnicity					
Indigenous	79	89.8	79	89.8	1.000^a^
Non-Indigenous	9	10.2	9	10.2	
Religion					
Catholic	30	34.1	36	40.9	.124^b^
Evangelical	41	46.6	29	33.0	
None	17	19.3	20	22.7	
Other (Maya)	0	0.0	3	3.4	
Education					
No education	20	22.7	18	20.5	.243^b^
Incomplete/ complete primary	60	68.1	57	64.8	
Incomplete/ complete secondary	5	5.7	10	11.4	
Incomplete/ complete tertiary	3	3.4	3	3.4	
Occupation					
Housewife	85	96.6	86	97.7	1.000^b^
Working	2	2.2	2	2.2	
Student	1	1.1	0	0.0	
Monthly income (GTQ)					
<1,000	20	22.7	15	17.0	.291^a^
1,000–1,999	26	29.5	20	22.7	
2,000–2,999	25	28.4	27	30.7	
3,000–3,999	9	10.2	14	15.9	
4,000–4,999	3	3.4	9	10.2	
≧5,000	5	5.7	3	3.4	
Marital status					
Cohabiting	49	55.7	58	65.9	.370^a^
Married	33	37.5	26	29.5	
Single	6	6.8	4	4.5	
Language spoken at home					
K'iche'	72	81.8	70	79.5	.583^b^
Spanish	16	18.2	16	18.2	
Achi	0	0.0	2	2.3	
Spanish language proficiency					
None/ only listen	26	29.5	39	44.3	.031^b^
Listen and speak	18	20.5	6	6.8	
Listen, speak and read	44	50.0	43	48.8	
Mode of birth					
Vaginal	37	42.0	45	51.1	.227^a^
Cesarean	51	58.0	43	48.9	
Experience of hospital birth					
Yes	35	39.8	44	50.0	.173^a^
No	53	60.2	44	50.0	

a Chi-square; ^b^Fisher's exact test.

### The primary outcome: women’s experiences of RMC

The mean PCMC score significantly increased from 33.74 (*SD* = 14.91) in the pre-intervention group to 56.70 (*SD* = 11.74) in the post-intervention group (*p* < .001), reflecting a relative increase of 68.0% (Table 2). All three subscale scores showed significant improvements, with the most notable relative increase of 97.7% observed in the communication and autonomy subscale, which rose from 7.01 to 13.86 (*p* < .001). A one-way ANCOVA, adjusting for factors related to PCMC, confirmed that post-intervention scores were significantly higher than pre-intervention scores (*p* < .001).

**Table 2:** Full and subscale person-centered maternity care (PCMC) scale scores.

Scale	Pre-intervention		Post-intervention		Change	*df*	*t*	*p*	Cohen's *d*
	*M*	*SD*	*M*	*SD*					
Full PCMC scale	33.74	14.91	56.70	11.74	68.0%	164	11.35	<.001	1.71
Dignity and respect (subscale)	9.39	3.52	14.81	2.75	57.1%	164	11.40	<.001	1.72
Communication and autonomy (subscale)	7.01	5.62	13.86	5.57	97.7%	173	8.12	<.001	1.23
Supportive care (subscale)	17.34	7.15	28.03	5.49	61.7%	163	11.13	<.001	1.68

**Figure 1:**
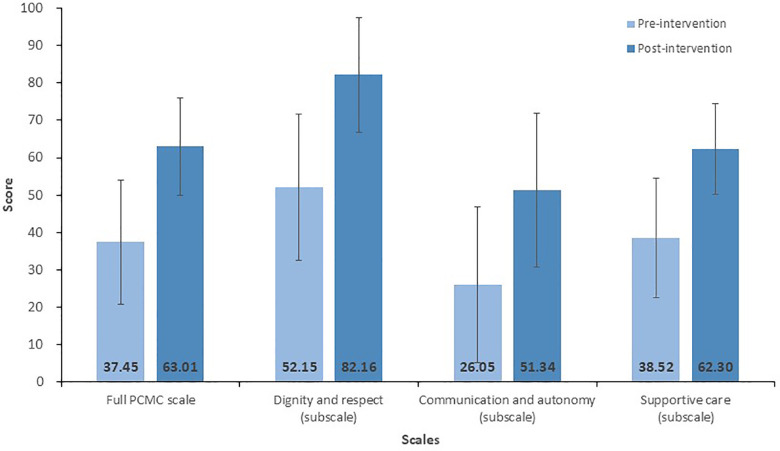
Rescaled full and subscale person-centered maternity care (PCMC) scale scores.

The PCMC scale and its subscales were converted to a 100-point scale for comparison within the subscales (Figure 1). The communication and autonomy subscale scored lowest before and after the intervention.

Table 3 presents the number and percentage of women in the pre- and post-intervention groups who answered “yes, most of the time” or “yes, all the time” to the items on the PCMC scale. Among the 30 items, 24 significantly increased in the post-intervention group. In the dignity and respect subscale, 5 out of 6 items showed significant improvement, with the percentage of women reporting being treated with respect increasing from 39.8% to 84.1% (*p* < .001). In the communication and autonomy subscale, 6 out of 9 items showed improvement, especially the percentage of women who were asked for permission or consent before procedures, which increased from 13.6% to 72.7% (*p* < .001). However, no significant changes were observed regarding nurses who introduced themselves and women being able to be in the position of their choice. In the supportive care subscale, 12 out of 15 items improved, with the percentage of women feeling safe in the hospital increasing from 36% to 84% (*p* < .001). At the same time, the proportion of women reporting that they were allowed to have someone of their choice stay with them during labor and delivery remained at 0% in both groups.

**Table 3:** Number and frequency of women who answered “yes, most of the time” or “yes, all the time” to the items on the person-centered maternity care (PCMC) scale.

Items	Pre-intervention		Post-intervention		*p*
	*n*	%	*n*	%	
Dignity and respect					
1. Did the nurses at the facility treat you with respect?	35	39.8	74	84.1	<.001^a^
2. Did the nurses at the facility treat you in a friendly manner?	41	46.6	69	78.4	<.001^a^
3. During examinations in the labor room, were you covered up?	10	11.4	38	43.2	<.001^a^
4. Do you feel like your health information was or will be kept confidential at this facility?	19	21.6	83	94.3	<.001^a^
5. Did you feel the nurses shouted at you, scolded, insulted, threatened, or talked to you rudely? [R]^c^	18	20.5	4	4.5	.001^a^
6. Did you feel like you were treated roughly like pushed, beaten, slapped, pinched, physically restrained, or gagged? [R]^c^	5	5.7	2	2.3	.444^b^
Communication and autonomy					
1. During your time in the health facility did the nurses introduce themselves to you when they first came to see you?^d^	4	4.5	7	8.0	.350^a^
2. Did the nurses call you by your name?	38	43.2	75	85.2	<.001^a^
3. Did you feel like the nurses at the facility involved you in decisions about your care?	12	13.6	33	37.5	<.001^a^
4. Did the nurses at the facility ask your permission or consent before doing procedures on you?	12	13.6	64	72.7	<.001^a^
5. During the delivery, do you feel like you were able to be in the position of your choice?	22	25.0	33	37.5	.074^a^
6. Did the nurses at the facility speak to you in a language you could understand?	23	26.1	63	71.6	<.001^a^
7. Did the nurses explain to you why they were doing examinations or procedures on you?	11	12.5	40	45.5	<.001^a^
8. Did the nurses explain to you why they were giving you any medicine?	19	21.6	35	39.8	.006^a^
9. Did you feel you could ask the nurses at the facility any questions you had?	9	10.2	32	36.4	<.001^a^
Supportive care					
1. How did you feel about the amount of time you waited? Would you say it was?^e^	41	45.6	66	75.0	<.001^a^
2. Did the nurses at the facility talk to you about how you were feeling?	14	15.9	39	44.3	<.001^a^
3. Did the nurses at the facility try to understand your anxieties?	8	9.1	19	21.6	.002^a^
4. Were you allowed to have someone you wanted (outside of staff at the facility, such as family or friends) to stay with you during labor?	0	0.0	0	0.0	1.000^b^
5. Were you allowed to have someone you wanted to stay with you during delivery?	0	0.0	0	0.0	1.000^b^
6. When you needed help, did you feel the nurses at the facility paid attention?	16	18.2	42	47.7	<.001^a^
7. Do you feel the nurses did everything they could to help control your pain?	16	18.2	66	75.0	<.001^a^
8. Do you think there were enough nurses in the facility to care for you?	41	46.6	61	69.3	.002^a^
9. Did you feel the nurses at the facility took the best care of you?	15	17.0	63	71.6	<.001^a^
10. Did you feel you could completely trust the nurses at the facility with regards to your care?	16	18.2	64	72.7	<.001^a^
11. Thinking about the labor and postnatal wards, did you feel the health facility was crowded?^f^	41	46.6	7	8.0	<.001^a^
12. Thinking about the wards, washrooms, and the general environment of the health facility, would you say the facility was very clean, clean, dirty, or very dirty?^g^	63	71.6	79	89.8	.002^a^
13. Was there water in the facility?	74	84.1	88	100.0	<.001^a^
14. Was there electricity in the facility?	86	97.7	88	100.0	.497^b^
15. In general, did you feel safe in the health facility?	32	36.4	74	84.1	<.001^a^

The number includes those who answered “Yes, all the time” or “Yes, most of the time.” R is reversed coded.

a Chi-square.

b Fisher's exact test.

c This includes the answers “No, never” and “Yes, once”.

d This includes the answers “Yes, all of them” and “Yes, most of them”.

e This includes the answers “very short” and “somewhat short”.

f This includes the answers “Yes, many times” and “Yes, a few times”.

g This includes the answers “Very clean” and “Clean”.

### The secondary outcome: women’s experiences of mistreatment

The percentage of women who experienced physical abuse, verbal abuse, or stigma or discrimination was 71.6% in the pre-intervention group. In contrast, in the post-intervention group, this percentage significantly decreased to 33.0% (*p* < .001). Table 4 presents the number and frequency of mistreatment experienced by women. Of the 26 items, 15 showed a decrease in mistreatment following the intervention, while 11 showed no statistically significant change. In the subcategories of verbal abuse, stigma and discrimination, and failure to meet professional standards of care, significant reductions were observed in nearly all items. In the verbal abuse subcategory, all items showed significant decreases, particularly the proportions of women reporting being threatened and scolded, which decreased from over 40% to 5.7% and 14.8%, respectively (*p* < .001). In the stigma and discrimination subcategory, all items showed significant decreases, with no women reporting discrimination based on age, education, or economic status, although discrimination based on race were still reported by two women post-intervention. In the failure to meet professional standards of care subcategory, all items showed a significant decrease, except for one: nurse absence at the baby's birth. Notably, significant reductions were observed in the lack of explanation and consent for procedures and inappropriate pain relief (*p* < .001).

**Table 4:** Number and frequency of mistreatment experienced by women.

Items	Pre-intervention		Post-intervention		*p*
	*n*	%	*n*	%	
Physical abuse					
Pinched	6	6.8	1	1.1	.118^b^
Slapped	4	4.5	0	0.0	.121^b^
Restrained to bed	5	5.7	0	0.0	.059^b^
Forceful downward pressure on abdomen	23	26.1	20	22.7	.599^a^
Verbal abuse					
Shouted at	27	30.7	7	8.0	<.001^a^
Scolded	36	40.9	13	14.8	<.001^a^
Negative comments about women's sexual activity	24	27.3	2	2.3	<.001^a^
Threatened with poor outcome	41	46.6	5	5.7	<.001^a^
Stigma and discrimination					
Discrimination based on race	15	17.0	2	2.3	<.001^a^
Discrimination based on age	14	15.9	0	0.0	<.001^a^
Discrimination based on education	8	9.1	0	0.0	.007^b^
Discrimination based on economic circumstances	7	8.0	0	0.0	.014^b^
Failure to meet professional standards of care					
No procedure explanation/ consent	55	62.5	18	9.1	<.001^a^
No vaginal exam conducted privately, so others could see	77	87.5	54	61.4	<.001^a^
No pain relief offered appropriately	62	70.5	13	14.8	<.001^a^
Neglected	54	61.4	26	29.6	<.001^b^
Waited long periods	47	53.4	31	35.2	.020^b^
Nurse absent when baby born	2	2.3	0	0.0	.497^b^
Poor rapport between women and providers					
No emotional support	38	43.1	8	9.1	<.001^b^
Not listened to about concerns	69	78.4	57	64.8	.058^b^
Birth companion not allowed	88	100.0	88	100.0	1.000
Not told could move around during labor	74	84.1	65	73.9	.096^a^
Health system conditions and constraints					
Lack of privacy/ curtains	73	83.0	63	71.6	.072^a^
No bed to self at postpartum	0	0.0	0	0.0	1.000
Bed share at any time	3	3.4	0	0	.246^b^
Asked for a bribe	0	0.0	0	0.0	1.000

a Chi-square.

b Fisher's exact test.

By contrast, no significant reductions were observed for most items within the subcategories of physical abuse, poor rapport between women and providers, and health system conditions and constraints, In the physical abuse subcategory, forceful downward pressure on the abdomen was experienced by more than 20% of women in both groups, with no significant change. In the poor rapport between women and providers subcategory, no significant decrease was observed, except for the item related to lack of emotional support. The item regarding the prohibition of birth companions remained unchanged at 100%. In the health system conditions and constraints subcategory, there was a slight reduction in the lack of privacy/curtains; however, this change was not statistically significant. There were no reports of the individual postpartum woman lacking a bed or requests for bribes.

## Discussion

This study aimed to evaluate the effectiveness of the RMC educational intervention for nurses. The results indicate that the educational intervention increased women's experiences of RMC and decreased their experiences of mistreatment. Specifically, the PCMC scores, which measured women's experiences of RMC, showed a significant increase of 68% after the intervention. This finding is consistent with a study conducted in Ghana, which reported a relative increase of 43% in the PCMC score (30). These results suggest that educational interventions effectively improve women's experiences of RMC. While the Ghana study utilized a two-day simulation training approach (30), this study employed a 6-hour interactive learning approach combining lectures and group work. Regardless of the educational format, both studies highlight the critical role of staff education in improving women's childbirth experiences.

Furthermore, the educational intervention in this study significantly reduced the percentage of women who experienced physical abuse, verbal abuse, or stigma or discrimination from 71.6% to 33.0%. This result aligns with previous research on multi-component interventions, including educational interventions, which reduced mistreatment in countries such as Tanzania, Ethiopia, and Kenya (37–40). Importantly, this study demonstrated a significant reduction in mistreatment using an educational intervention alone, reinforcing the critical role of education as a powerful and effective tool for reducing mistreatment during childbirth. Among Indigenous women in Guatemala, respectful care from healthcare providers has been strongly associated with satisfaction during childbirth (21). This further emphasizes that providing RMC is particularly important in this region. Mistreatment is driven by factors at various levels, including individual, facility, and policy levels, and therefore, comprehensive interventions at multiple levels are necessary (41). Facility and policy reforms require time and resources, whereas educational interventions are relatively low-cost, quick to implement, and practical, especially in resource-limited settings. Enhancing healthcare providers’ perceptions and attitudes can lead to immediate improvements; therefore, these interventions should be prioritized for implementation.

While improvements following the intervention were observed in this study, it also suggests the need for continued efforts to promote RMC in other regions. This study's pre-intervention PCMC scores were lower than those reported in previous studies conducted in Africa and Asia (30, 34, 42, 43). In addition, the incidence of mistreatment was higher compared with studies conducted in four countries (2). Research has indicated that younger women, unmarried women, women with lower educational levels, and ethnic minorities are more likely to experience mistreatment (1, 2, 44). Moreover, factors such as marital status, education level, socioeconomic status, and the type of healthcare facility influence PCMC scores (33, 43). In the region where this study was conducted, 90% of the population is Indigenous, 86% live in poverty, and the literacy rate is 31% (17, 45). The participants in this study also reflected the region's characteristics, with 90% identifying as Indigenous, approximately 50% being literate, and most households having a monthly income below the rural average of 5,368.79 GTQ, as reported in national statistics (46). Given these sociodemographic characteristics, the women in this study likely had many risk factors, which, combined with the fact that this study was conducted in a secondary healthcare facility, may help explain the lower PCMC scores and higher levels of mistreatment observed. Although research on RMC in Latin America is limited (10–12), regions with sociodemographic characteristics similar to this study area in Guatemala are widely distributed both within Guatemala and across Latin America. Therefore, implementing initiatives to promote RMC in these regions is required.

The intervention in this study suggested improvements in women's childbirth experiences. However, challenges remain in areas such as rapport and communication between women and healthcare providers, and women's autonomy. This suggests that continuous education and further efforts are needed to establish RMC as a standard of care. Like the findings of this study, research conducted in four countries across Asia and Africa has also highlighted the lack of communication and autonomy as challenges (2). In situations where mistreatment occurs, there are reports of the differences in position between healthcare providers and women, as well as the authoritarian attitudes of providers (1, 47). In Guatemala, it is documented that women felt they must obey healthcare providers (23), and such authoritarian perceptions create an imbalanced power dynamic between women and providers, hindering the provision of person-centered care. Therefore, while continuous education is essential to change healthcare providers’ perceptions, it is also necessary to establish systems that enable the standard practice of such care without solely relying on changes in providers’ attitudes. Organizational responses, including protocol development and staff supervision, are required to provide high-quality care (48). Furthermore, continuous efforts and long-term follow-up are needed to establish RMC.

The results of this study suggest that education for all healthcare staff is crucial for achieving RMC. In this study, the educational intervention was limited to nurses, with no education provided for physicians or other healthcare providers. This may explain the limited improvements observed in several items. Given that deliveries in the facility were assisted by physicians, the lack of progress in areas such as “forced downward abdominal pressure”, “mobility during labor”, and “no vaginal exam conducted privately” was to be expected. Previous studies emphasize the importance of involving all staff in education (49, 50). Mistreatment is perpetrated not only by nurses, midwives, and physicians but also by non-medical staff, such as receptionists (44), highlighting the need for education among non-medical staff. Therefore, to effectively promote RMC, it is essential to educate not only healthcare providers directly involved in women's care but also all hospital staff.

This study also suggests that improvements at both the facility and policy levels are crucial for establishing RMC. Improvements such as “delivery in the preferred position”, “companionship during delivery”, and “lack of privacy/curtains” require not only educational interventions but also changes in facility policies and infrastructure. However, in public facilities, many issues cannot be addressed solely at the facility level, making national-level support and policy reinforcement indispensable. The Ministry of Public Health and Social Assistance in Guatemala recommends these elements as part of culturally appropriate healthcare services (51). However, the findings of this study revealed that women were forced to deliver in the lithotomy position and the presence of a companion during labor was not permitted. The lack of necessary infrastructure and staff has created a gap between policy and healthcare practice. In contrast, interventions led by the Ministry of Health and healthcare institutions in Ethiopia have been reported as successful in promoting the presence of companions during labor (52, 53). Based on these findings, establishing RMC requires facility- and national-level policy support and reinforcement.

### Limitations

This study represents the first attempt to implement an educational intervention on RMC in Guatemala. Consistent with findings in other countries, the educational intervention in Guatemala was suggested to contribute to improvements in women's childbirth experiences. However, this study has some limitations. First, this study was conducted in a single site, limiting the generalizability of the results. Therefore, future research should include multiple facilities and incorporate methods such as cluster-randomized controlled trials to provide a more comprehensive and reliable evaluation of the effects of the educational intervention. Second, due to the characteristics of the study area, self-administered questionnaires were not feasible; therefore, interviews were also employed. Although the study purpose was carefully explained and rapport established prior to data collection, conducting the interviews within the facility means that the possibility of social desirability bias cannot be ruled out. Lastly, due to time constraints, women's childbirth experiences were assessed only once immediately after the intervention, and long-term effects were not evaluated. Future research should include long-term follow-ups to assess whether the impact of the intervention is sustained over time.

## Conclusion

The findings of this study suggest that educational interventions may have a positive effect on improving women's childbirth experiences in healthcare facilities. Therefore, expanding these educational programs to other facilities and regions could contribute significantly to promoting RMC. Furthermore, it is essential to implement the program for all staff working at the facility. In addition to educational interventions, comprehensive initiatives at the facility and policy levels are essential for establishing RMC.

## Data Availability

The raw data supporting the conclusions of this article will be made available by the authors, without undue reservation.
